# Modeling influenza epidemics and pandemics: insights into the future of swine flu (H1N1)

**DOI:** 10.1186/1741-7015-7-30

**Published:** 2009-06-22

**Authors:** Brian J Coburn, Bradley G Wagner, Sally Blower

**Affiliations:** 1 Biomedical Modeling Center, Semel Institute of Neuroscience & Human Behavior, David Geffen School of Medicine at UCLA, Los Angeles, CA, USA

## Abstract

Here we present a review of the literature of influenza modeling studies, and discuss how these models can provide insights into the future of the currently circulating novel strain of influenza A (H1N1), formerly known as swine flu. We discuss how the feasibility of controlling an epidemic critically depends on the value of the Basic Reproduction Number (*R*_0_). The *R*_0 _for novel influenza A (H1N1) has recently been estimated to be between 1.4 and 1.6. This value is below values of *R*_0 _estimated for the 1918–1919 pandemic strain (mean *R*_0_~2: range 1.4 to 2.8) and is comparable to *R*_0 _values estimated for seasonal strains of influenza (mean *R*_0 _1.3: range 0.9 to 2.1). By reviewing results from previous modeling studies we conclude it is theoretically possible that a pandemic of H1N1 could be contained. However it may not be feasible, even in resource-rich countries, to achieve the necessary levels of vaccination and treatment for control. As a recent modeling study has shown, a global cooperative strategy will be essential in order to control a pandemic. This strategy will require resource-rich countries to share their vaccines and antivirals with resource-constrained and resource-poor countries. We conclude our review by discussing the necessity of developing new biologically complex models. We suggest that these models should simultaneously track the transmission dynamics of multiple strains of influenza in bird, pig and human populations. Such models could be critical for identifying effective new interventions, and informing pandemic preparedness planning. Finally, we show that by modeling cross-species transmission it may be possible to predict the emergence of pandemic strains of influenza.

## Introduction

Mathematical models have been used to understand the spatial-temporal transmission dynamics of influenza. They have also been used as health policy tools to predict the effect of public health interventions on mitigating future epidemics or pandemics. The potential epidemiological impact of both behavioral and biomedical interventions has been investigated. Here we present a review of the literature of influenza modeling studies and discuss how results from these studies can provide insights into the future of the currently circulating strain of novel influenza A (H1N1). This strain was formerly known as swine flu [1].

## A basic epidemiological model for Influenza

**Figure 1:**
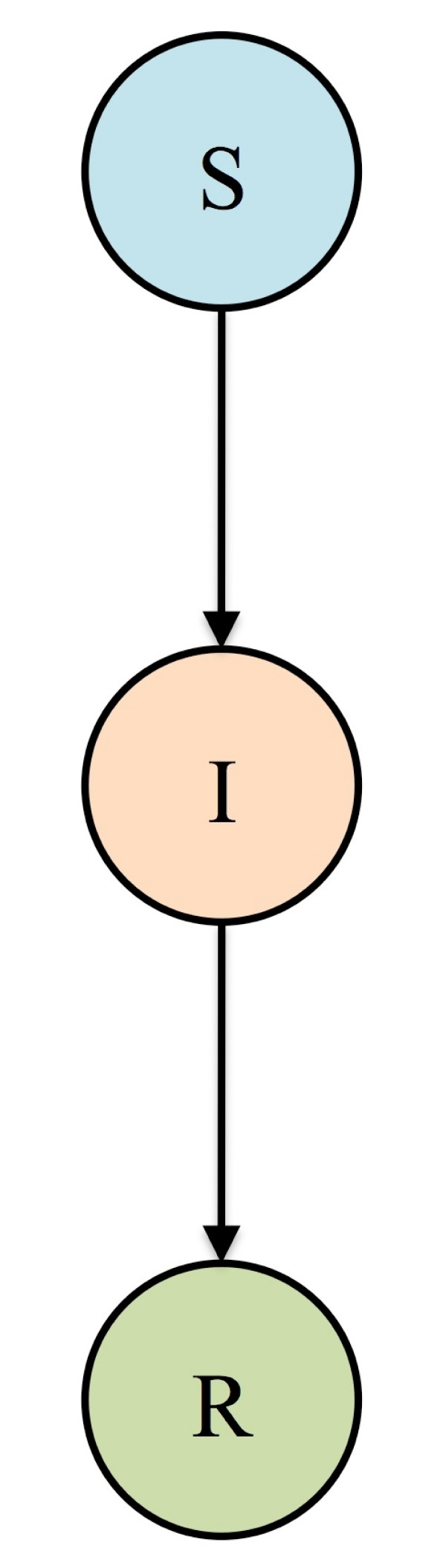
**Compartmental SIR model of disease transmission**. The population is partitioned into three classes: Susceptible (S), Infectious (I), and Recovered (R). Individuals who become infected proceed from class S to class I at a rate which depends on the infectiousness of the virus and the prevalence of infection. Infectious individuals recover and move to class R, at which point they are immune to future infection. The model can be straightforwardly extended to include immunity which wanes over time.

The first mathematical model that could be used to describe an influenza epidemic was developed early in the 20th century by Kermack and McKendrick [2]. This model is known as the Susceptible-Infectious-Recovered (SIR) model, and is shown as a flow diagram in Figure 1. To simulate an influenza epidemic the model is analyzed on a computer and one infected individual (I) is introduced into a closed population where everyone is susceptible (S). Each infected individual (I) transmits influenza, with probability *β*, to each susceptible individual (S) they encounter. The number of susceptible individuals decreases as the incidence (i.e., the number of individuals infected per unit time) increases. At a certain point the epidemic curve peaks, and subsequently declines, because infected individuals recover and cease to transmit the virus. Only a single influenza epidemic can occur in a closed population because there is no inflow of susceptible individuals. The severity of the epidemic and the initial rate of increase depend upon the value of the Basic Reproduction Number (*R*_0_). *R*_0 _is defined as the average number of new infections that one case generates, in an entirely susceptible population, during the time they are infectious. If *R*_0 _> 1 an epidemic will occur and if *R*_0 _< 1 the outbreak will die out. The value of *R*_0 _for any specific epidemic can be estimated by fitting the SIR model to incidence data collected during the initial exponential growth phase. The value of *R*_0 _may also be calculated retroactively from the final size of the epidemic. If the SIR model is used, *R*_0 _for influenza is equal to the infectivity/transmissibility of the strain (*β*) multiplied by the duration of the infectious period. Therefore once the value of *R*_0 _has been obtained, the value of β can be determined.

**Figure 2:**
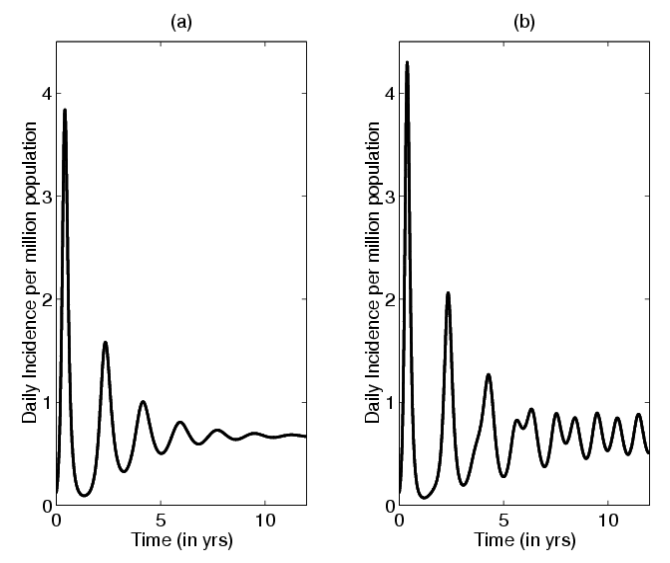
**Daily incidence for an influenza outbreak calculated using an SIR model**. **(a) **With constant transmission rate. **(b) **With small seasonal variation in transmission rate. For a constant transmission rate, after an initial transient period, the system approaches an endemic level (that is, equilibrium) by damped oscillations. If a small amount of seasonal variation in transmission is introduced oscillations are sustained rather than damping out, and the system eventually tends to an annual cycle.

The SIR model has been used as a basis for all subsequent influenza models. The simplest extension to the SIR model includes demographics; specifically, inflow and outflow of individuals into the population. Analysis of this demographic model shows that influenza epidemics can be expected to cycle, with damped oscillations, and reach a stable endemic level (Figure 2A). By modifying the basic SIR model in a variety of ways (e.g., by including seasonality [3,4]) influenza epidemics can be shown to have sustained cycles (Figure 2B). The SIR model has also been extended so that it can be used to represent and/or predict the spatial dynamics of an influenza epidemic. The first spatial-temporal model of influenza was developed in the late 1960s by Rvachev [5]. He connected a series of SIR models in order to construct a network model of linked epidemics. He then modeled the geographic spread of influenza in the former Soviet Union by using travel data to estimate the degree of linkage between epidemics in major cities. In the 1980s, he and his colleagues Baroyan and Longini extended his network model and evaluated the effect of air travel on influenza pandemics [6,7]. Since then other modeling studies have quantified the importance of air travel on geographic spread [8,9]. For example, a recent study has modeled the potential for influenza epidemics to move through nine European cities: Amsterdam, Berlin, Budapest, Copenhagen, London, Madrid, Milan, Paris, and Stockholm [8]. The authors estimate that, due to a high degree of connectedness through air travel, it would take less than a month for an epidemic beginning in any one of these cities to spread to the other eight [8]. Network models have also been use to understand the temporal and spatial synchrony of influenza epidemics within the United States (US) [10].

## Modeling the past

Modeling studies have provided interesting insights into the severity of past influenza epidemics and pandemics [11-15]. For example, Chowell and colleagues have compared the severity of seasonal influenza epidemics in the US, France, and Australia over the past three decades by estimating country-specific values of *R*_0 _[11]. Their results show the severity of the epidemics in the three countries is similar every year, but there is considerable year to year variability (mean *R*_0 _is 1.3; range is 0.9 to 2.1) [11]. Many modeling studies have investigated the three historical pandemics of the 20th century: the Spanish Flu 1918–1919 (H1N1), Asian Flu 1957–1958 (H2N2), and Hong Kong Flu 1968 (H3N2) [7,12-17]. Mills *et al.*, using pneumonia and influenza mortality data collected in 45 cities in the USA, estimated that the value of *R*_0 _for the 1918–1919 pandemic was between 2 and 3 [14]. Ferguson *et al. *reached similar conclusions; they estimated that an *R*_0_~2 with a range of 1.4 to 2.8 [18]. Modeling has also been applied to assess the effect that interventions may have had in mitigating the 1918–1919 pandemic [12,17]. Bootsma *et al. *[12] estimated that public health measures, based on social distancing, reduced mortality by 10 to 30% in cities in the US. They concluded that the timing of public health interventions strongly influenced the magnitude of the autumn wave of influenza [12]. Another study used data on daily mobility patterns of fur traders traveling between settlements, and modeled the effectiveness of voluntary quarantine on the spread of influenza in central Canada during the 1918–1919 pandemic [17]. The authors found that, as mobility rates were low, only extremely high rates of quarantine would have significantly altered the pattern of geographic spread [17].

## Designing biomedical and behavioral public health interventions

**Figure 3:**
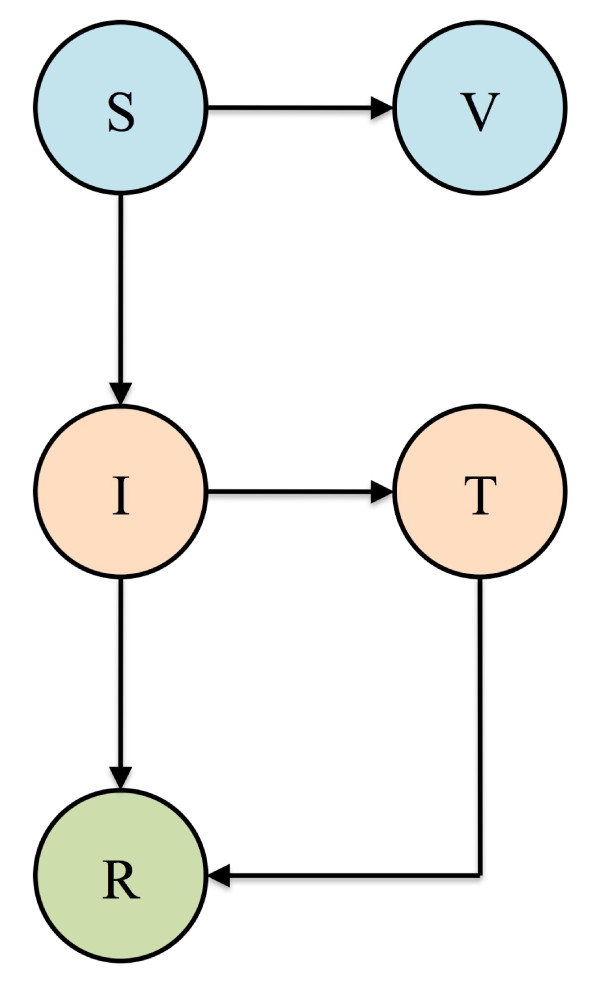
**SIR compartmental model of disease transmission incorporating vaccination and treatment**. Susceptible individuals (S) who are vaccinated proceed to class V, at which point they are considered immune. Upon treatment, infectious individuals (I) proceed to class T, at which point their infectiousness is reduced.

**Figure 4:**
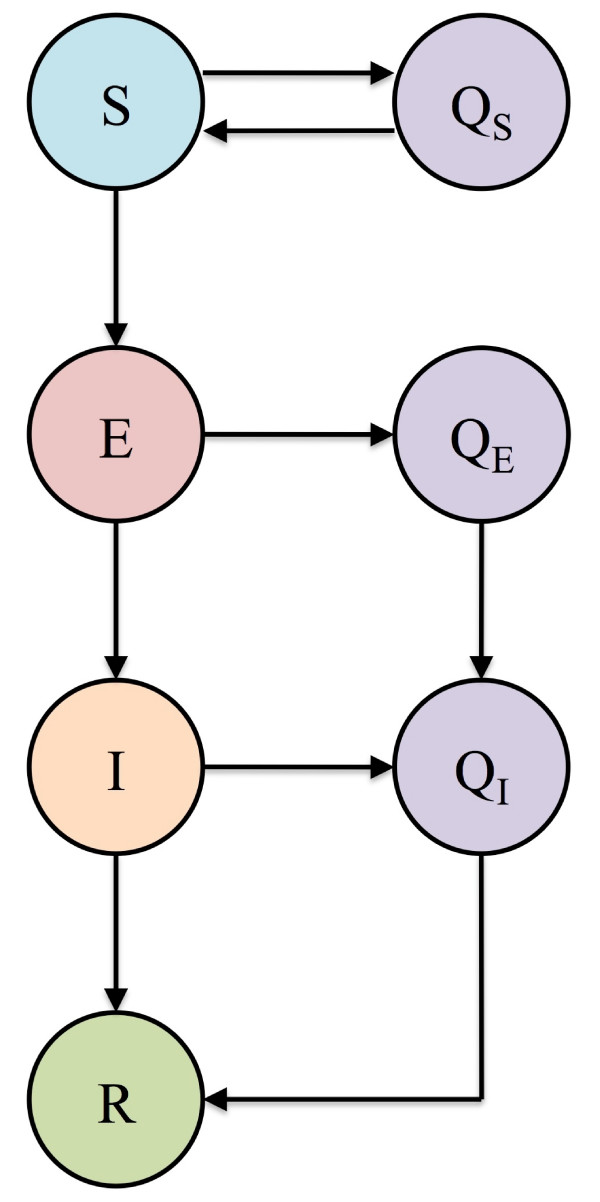
**SEIR compartmental model of infection incorporating quarantine and isolation measures**. The class E represents a latent class during which an individual who has been exposed to the pathogen is not yet infectious and is asymptomatic. Individuals who might have been exposed (S and E) are quarantined and proceed to the respective Q classes: Q_S _and Q_E_. When susceptible individuals in quarantine (Q_S_) are determined not to have been infected they are returned to the susceptible class (S). Those in quarantine who develop infection (Q_E_) are isolated and proceed to class (Q_I_), as do infected individuals (I).

Behavioral and biomedical interventions have been modeled using relatively simple extensions of the SIR model (as shown in Figures 3 and 4[19]) or by implementing the SIR model within a framework of a detailed simulation model [10]. Figure 3 shows an extension of the SIR model that includes two biomedical interventions: susceptible individuals (S) can be vaccinated and infected individuals (I) can be treated with antiviral drugs (T). Figure 4 shows an extension that includes two behavioral interventions: quarantine and isolation. Susceptible individuals (S) can be quarantined (Q_S_) and then returned to the pool of susceptible individuals (S) once it is determined they are uninfected. In addition, infected, asymptomatic and not yet infectious individuals (E) can be quarantined (Q_E_). If they develop symptoms and become infectious they can be isolated (Q_I_), as can the infected individuals (I) (Figure 4). Once interventions have been included in the model the Reproduction Control Number (*R*_*C*_) can be determined. *R*_*C *_is defined as the average number of new infections that one case generates, in an entirely susceptible population when an intervention is in place, during the time they are infectious. The value of *R*_*C *_will depend on both the strength of the intervention and the severity of the epidemic in the absence of the intervention (*R*_0_). *R*_*C *_will always be less than *R*_0_, but if *R*_*C *_< 1 the intervention will cause the epidemic to die out, whereas if *R*_*C *_> 1 the intervention will only reduce the severity of the epidemic.

The effect of behavioral interventions such as closing schools, quarantining infected individuals or imposing travel restrictions have been modeled [17,18,20-23]. It has been shown that behavioral interventions that increase social distancing, such as prolonged school closures, could reduce the cumulative number of influenza cases by 13 to 17% [20]. Studies have been useful for comparing interventions. For example, Ferguson *et al*. have determined that household quarantine could be more effective than closing schools [18]. The potential effectiveness of biomedical interventions (for example, vaccination, prophylactic treatment with antivirals, and therapeutic treatment) have been modeled [19,24,25]. Models have also been used to compare the relative effectiveness of prophylaxis versus treatment strategies [24], to assess the potential problem of antiviral resistance [26-31] and to identify the optimal strategy for allocating vaccines [25]. Most studies have evaluated the potential effectiveness of a combination of behavioral and biomedical interventions [18,22,23,32]. Some studies have shown that certain interventions are unlikely to be effective. For example, Cooper *et al.*, Ferguson *et al.*, and Epstein *et al. *have found that even extensive air travel restrictions would be unlikely to delay spread of a pandemic by more than a few weeks [18,21,33]. However, Colizza *et al. *have shown that a pandemic could be effectively contained if there is a global cooperative strategy in place, whereby one country donates some of their stockpiled antivirals to other countries in need [34]. Not surprisingly, all of the studies have shown it is essential to implement interventions as quickly and as early in the epidemic as possible.

## Feasibility of biomedical and behavioral public health interventions

Although many studies have identified potentially effective public health interventions, they have not assessed their feasibility. For example, studies evaluating mass vaccination strategies have found a very high coverage is needed to prevent epidemics. However, in the 'real-world' where vaccination is voluntary, high vaccination coverage is rarely achieved. Recently Vardavas *et al. *and Galvani *et al. *have investigated the effect of human behavior on determining vaccination coverage [35,36]. Vardavas *et al. *constructed a dynamic individual-level model of human cognition and behavior; individuals in this model are characterized by two biological attributes (memory and adaptability) they use when making vaccination decisions [35]. Individuals are allowed to decide, on the basis of self-interest, whether to vaccinate or not each year. In addition, individuals are given an option of changing their vaccination behavior each year. Consequently, individual-level adaptive behavior influences influenza epidemiology, and conversely, influenza epidemiology influences individual-level vaccination decisions. Galvani *et al. *took a different approach and developed a static model based on Game Theory [36]. Both Vardavas *et al*. and Galvani *et al. *showed that coverage levels high enough to achieve herd immunity could only be attained by implementing incentive-based vaccination programs [35,36]. However, Vardavas *et al*. also showed that certain of these programs could, paradoxically, increase epidemic severity [35]. They therefore recommend incentive-based vaccination programs will need to be very carefully designed [35]. The studies of Vardavas *et al. *and Galvani *et al. *illustrate that models can be used to identify the strength of the interventions that are necessary to control an epidemic or pandemic, but the goals of the control strategy may not be attainable.

The feasibility of controlling an epidemic will critically depend on the value of the R_0_. The more severe the epidemic (i.e., the greater the value of R_0_) the more intensive the interventions must be to significantly reduce the number of infections and deaths. Not surprisingly, the levels of vaccination or treatment necessary for control are lower if interventions are targeted. For example, Longini *et al. *modeled the effects of age-specific targeting strategies and found vaccinating 80% of children (less than 19 years old) would be almost as effective as vaccinating 80% of the entire population [24]. Longini *et al. *also found that targeting antiviral prophylaxis (that is, providing close contacts of suspected cases with antivirals) could be extremely effective in controlling epidemics [24]. However even using a targeted approach they determined it would be necessary for 80% of exposed individuals to be quickly identified, and for them to take antivirals for up to 8 weeks in order to mitigate a severe epidemic [24].

Many studies have evaluated the level of interventions needed to contain epidemics of varying severity. For example, Colizza *et al. *simulated a hypothetical influenza pandemic that was capable of spreading through 3,100 urban areas in 220 countries [34]. When *R*_0_was less than 1.9 they found the epidemic could be significantly reduced if there were enough antivirals to treat ~2–6% of the population. However, when they modeled a very severe epidemic (*R*_0 _of 2.3) their simulations showed, that even if ~20% of the population were treated with antivirals, 30–50% of the population would become infected [34]. Longini *et al. *conducted a similar type of analysis, but assessed the potential for interventions to control an emerging influenza epidemic in rural South East Asia [22]. They determined that targeted antiviral prophylaxis could contain a moderately severe epidemic (*R*_0 _< 1.6) if 100,000 to 1 million courses of antivirals were available. A combination of targeted antiviral prophylaxis and pre-epidemic vaccination would be necessary to contain a severe epidemic (*R*_0_~2.1). Finally, they calculated that a combination of high levels of targeted antiviral prophylaxis, pre-vaccination, and quarantine could contain even a very severe epidemic (*R*_0_~2.4). Ferguson *et al. *also modeled an emerging influenza epidemic in South East Asia [32]. Their results show geographically targeted prophylaxis, reinforced with behavioral interventions aimed at increasing social distancing, would be necessary to contain an epidemic with an *R*_0 _of ~*1.6*. They calculated that 3 million courses of antivirals would be needed for their proposed control strategy.

The results from all of the modeling studies are in agreement; very high vaccination and treatment levels will be necessary to contain even a moderately severe pandemic. It will be difficult, but perhaps possible, to achieve these goals for interventions in resource-rich countries. However, clearly resource-constrained and resource-poor countries will be unable to achieve these goals unless they are given very large supplies of vaccines and antivirals by resource-rich countries.

## Modeling Influenza A (H1N1): emergence and control

Influenza is a zoonotic disease that can infect a variety of host species. Strains can be transmitted between species, and new strains can emerge through co-infection and genetic recombination in intermediate hosts. Wild ducks and wading birds are considered to be a reservoir for influenza because they can carry all subtypes, and the virus is avirulent to its avian hosts. Avian viruses are also found in other birds such as domestic ducks and poultry. New strains of avian influenza have recently emerged in South East Asia and have infected humans. These strains are not transmissible from human to human; however, they are highly virulent in humans and have killed approximately 70% of infected individuals [37]. Besides humans, avian influenza viruses infect a variety of other mammals including seals, whales, and pigs [38]. Considerable attention has been focused on avian influenza as it has been expected that pandemic strains would arise from transmission from birds to humans. However, surprisingly, influenza A (H1N1) emerged through cross-species transmission from pigs to humans and has been shown to have arisen due to recombination between swine, avian, and human strains.

The first modeling paper on influenza A (H1N1) has recently been published [39]. By fitting an SIR model to initial outbreak data from La Gloria in Mexico Fraser *et al*. estimated the *R*_0 _for this novel strain to be between 1.4 to 1.6 [39]. This value is on the lower end of previous values for the 1918–1919 strain (*R*_0 _mean ~2: range 1.4 to 2.8 [18]) and is comparable to *R*_0 _values estimated for seasonal strains of influenza (*R*_0 _mean 1.3: range 0.9 to 2.1 [11]). (It is important to note that there is considerable overlap in the estimates of *R*_0 _for seasonal and pandemic strains.) The public health measures that were widely applied in Mexico appear to have been successful in mitigating the outbreak of H1N1; this observation appears to corroborate results from earlier modeling studies [23] that show behavioral interventions can be very effective if R_0 _is below two. The *R*_0 _results of Fraser *et al*. from La Gloria (*R*_0 _for H1N1 lies between 1.4 and 1.6) indicate that it is theoretically possible to control this pandemic. However, as we have discussed previously, an effective control strategy that has been identified by modeling may not be a feasible control strategy. If a vaccine is available by the autumn there is likely to be high uptake, due to the publicity surrounding the initial outbreak of this strain. If the initial estimates of the R_0 _for H1N1 are correct then this high vaccination coverage could have a significant effective on mitigating the pandemic, at least in resource-rich countries. However, H1N1 has now been disseminated worldwide through air travel. Consequently, it will be necessary for resource-rich countries to share vaccines and antivirals in order to mitigate a pandemic. Such a global cooperative strategy will be essential to prevent resource-constrained and resource-poor countries suffering from a significantly disproportionate burden of morbidity and mortality.

**Figure 5:**
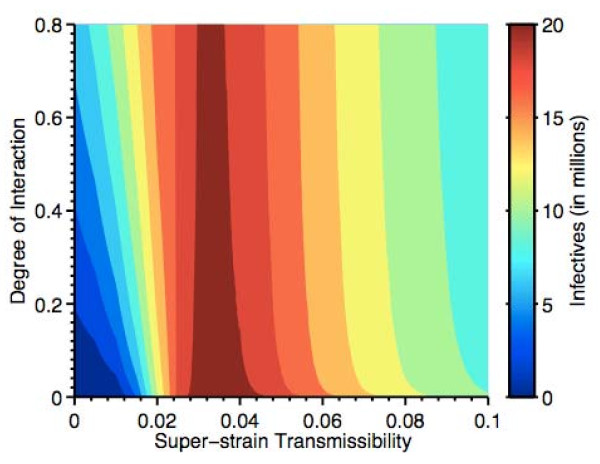
**Results from a cross-species multi-strain transmission model where pigs act as 'mixing vessels'**. This figure shows the severity of the epidemic that occurs when a 'super-strain' emerges into the human population from pigs, as a function of the cross-species interaction and the transmissibility/infectivity of the 'super-strain' in humans. The maximal number of infectives that could occur in any year (estimated over a 150-year time period) is shown in dark red. The contour map illustrates that the greatest outbreak occurs when the transmissibility/infectivity of the 'super-strain' is greater than 0.024 and less than 0.04; this implies 2.3 <*R*_0 _< 3.8.

Fraser *et al*. modeled the transmission dynamics of influenza A (H1N1) in the human population, but did not include cross-species transmission [39]. The emergence of H1N1 has shown the necessity for developing more biologically complex models that can provide a comprehensive understanding of strains that arise due to cross-species transmission. Coburn has recently developed one such complex model that tracks influenza transmission dynamics within three species (birds, pigs, and humans), as well as between these species [40]. His model includes several species-specific strains that infect birds, pigs, and humans. He models pigs as 'mixing vessels' which can be co-infected with avian, swine, and human strains of influenza. Species-specific strains can then undergo recombination in infected pigs and generate 'super-strains' that can be transmitted from pigs to humans. Analysis of his model generates significant insights into understanding the emergence of novel recombinant strains of influenza (such as H1N1), as well as in predicting their epidemic and pandemic potential. Surprisingly, his results show that an epidemic with an intermediate value of *R*_0 _could result in significantly more infected individuals than an epidemic with a high value of *R*_0_; see Figure 5 (the value of the transmissibility of the 'super-strain' in humans corresponds to the value of the *R*_0_). Specifically, the contour map in Figure 5 illustrates that the greatest outbreak occurs when the transmissibility/infectivity of the "super-strain" is greater than 0.024 and less than 0.04; this implies 2.3 < R_0 _< 3.8. In addition, Coburn's modeling shows that at low values of *R*_0 _the number of individuals that become infected will be very dependent on the degree of interaction between humans and pigs (Figure 5). Coburn's results illustrate how, by modeling cross-species transmission and determining the degree of interaction between pigs and humans, it may be possible to predict the emergence of pandemic strains of influenza.

To the best of our knowledge there are only two published studies that have modeled interventions for influenza strains that arise due to cross-species transmission. Iwami *et al. *modeled epidemics that result as a consequence of cross-species (that is, avian-human) transmission [41]. Their results show the potential effectiveness of quarantine as a control strategy, and also the importance of simultaneously controlling influenza in the avian population [41]. Saenz *et al. *modeled the potential effect of pigs (or poultry) on amplifying the number of infections that would arise as the result of a new strain of influenza [42]. They modeled the transmission dynamics in a confined feeding operation (CAFO) as a result of interactions between three groups: CAFO species (either swine or poultry), CAFO workers, and the rest of the local population. Their results show that amplification would be prevented if at least 50% of the CAFO workers could be successfully vaccinated [42]. They suggest that a vaccination strategy targeted at CAFO workers could be an effective strategy for containing a pandemic. Notably, the interventions suggested by Iwami *et al. *[41] and Saenz *et al. *[42] are interventions that cannot be identified unless cross-transmission is included in the model.

## Summary and conclusion

As we have discussed in this review, mathematical models have been extremely useful in increasing our understanding of the spatial-temporal transmission dynamics of influenza. They have also provided assistance in evaluating the potential effectiveness of public health interventions in controlling pandemics of varying severity, where severity has been defined by the value of *R*_0_. However, we have stressed that, although many theoretical interventions have been identified they may not be feasible. Furthermore, we have argued that pandemic control will only be attainable with a global cooperative strategy. Our review has shown that current models may not be useful in identifying effective interventions for epidemics generated by strains, such as influenza A (H1N1), that emerge due to recombination of species-specific strains and subsequent cross-species transmission. Therefore, we recommend that more biologically complex models need to be developed. Analysis of such models could assist in identifying interventions that would be effective in reducing the probability of cross-species transmission and in mitigating pandemics driven by multi-species transmission. Results from these new policy models could provide critical insights for informing pandemic preparedness planning.

## Abbreviations

CAFO: contained feeding operation; SIR: Susceptible-Infectious-Recovered.

## Competing interests

The authors declare that they have no competing interests.

## Authors' contributions

BJC, BGW, and SB conceived the study, carried out the literature review, interpreted the data and wrote the paper. BGW carried out the simulations for Figure 2 and BJC carried out the simulations for Figure 5.

## Pre-publication history

The pre-publication history for this paper can be accessed here:


